# Congenital Vomer Agenesis: A Rare and Poorly Understood Condition Revealed by Cone Beam CT

**DOI:** 10.3390/diagnostics8010015

**Published:** 2018-02-10

**Authors:** David Jun Yan, Vincent Lenoir, Sibylle Chatelain, Salvatore Stefanelli, Minerva Becker

**Affiliations:** 1 Department of Imaging and Informatics Sciences, Division of Radiology, University Hospital Geneva, University of Geneva, 1211 Geneva, Switzerland; DavidJun.Yan@hcuge.ch (D.J.Y.); Salvatore.Stefanelli@hcuge.ch (S.S.); Minerva.Becker@hcuge.ch (M.B.); 2 Division of Oral Surgery and Implantology, School of Dental Medicine, University of Geneva, 1211 Geneva, Switzerland; chatelainsibylle@gmail.com

**Keywords:** vomer agenesis, cone-beam computed tomography, primary failure of tooth eruption

## Abstract

Isolated congenital vomer agenesis is a very rare and poorly understood condition. In the context of dental work-up by cone-beam computed tomography (CBCT), the explored volume of the facial bones occasionally reveals incidental abnormalities. We report the case of a 13-year old Caucasian female who underwent CBCT for the pre-treatment evaluation of primary failure of tooth eruption affecting the permanent right upper and inferior molars. CBCT depicted a large defect of the postero-inferior part of the nasal septum without associated soft tissue abnormality and without cranio-facial malformation or cleft palate. In the absence of a history of trauma, chronic inflammatory sinonasal disease, neoplasia and drug abuse, a posterior nasal septum defect warrants the diagnosis of vomer agenesis. A discussion of this condition and of salient CBCT features is provided.

## 1. Introduction

The nasal septum is composed of the crests of the maxillary and palatine bones, the perpendicular plate of the ethmoid bone, the septal nasal cartilage and the vomer [1,2]. The vomer is an unpaired facial bone that connects the superior part of the nasal septum to the hard palate.

Isolated nasal septum defects have a variety of causes, such as nasal surgery or trauma (including iatrogenic occasional nose picking), infection (e.g., tuberculosis, syphilis, fungal infections, others), chronic inflammatory diseases (Wegener’s granulomatosis, sarcoidosis, autoimmune diseases), neoplasia (carcinoma, T-cell lymphoma) or drug abuse (e.g., cocaine, topical corticosteroids, krokodil drug, others) [3]. These pathologic conditions typically affect the antero-inferior part of the nasal septum. Among the etiologies of nasal septum defects, congenital agenesis of the vomer is a rare cause that typically affects the postero-inferior part of the nasal septum [4].

To the best of our knowledge, only about twenty cases of vomer agenesis (VA) have been reported in the medical literature so far, all of which were incidentally discovered by nasal endoscopic examination [4,5,6,7,8,9,10,11]. We report the case of a 13-year old girl with congenital vomer agenesis incidentally discovered on cone-beam computed tomography (CBCT) obtained for evaluation of primary failure of tooth eruption.

## 2. Case Report 

A 13-year-old Caucasian female was referred to our department for a CBCT examination to evaluate the relationship of the permanent right upper and lower molars with primary failure of eruption and the right maxillary sinus and right mandibular canal, respectively. There was no history of nasal surgery or trauma, and no drug abuse. The patient didn’t complain of any ear, nose and throat (ENT) symptoms such as sore throat, cough, nasal obstruction or posterior nasal dripping. No headache in the frontal region was mentioned. There was no velopharyngeal insufficiency.

**Figure 1:**
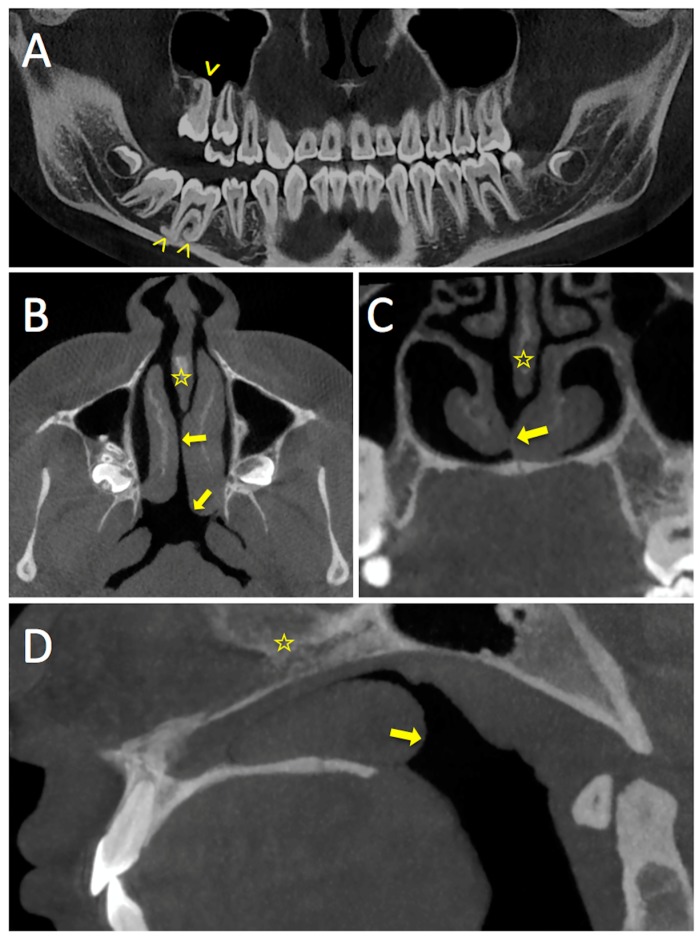
Multiplanar reconstruction of the CBCT acquisition in the curved panoramic-like plane (**A**) and in the horizontal (**B**), coronal (**C**), and sagittal planes (**D**). Panoramic-like reconstruction shows the hooked aspect of the root apices of teeth 16 and 46 (arrowheads) with primary failure of eruption. The nasal septum is incomplete (yellow stars) with a large defect in its postero-inferior part corresponding to complete absence of the vomer. The bony structures of the middle and lower turbinates appear to be preserved. Nevertheless, the left lower turbinate (arrows) is hypertrophied and protrudes through the septal defect. Note contact between the left and right lower turbinate and posterior protrusion of the left lower turbinate into the nasopharynx.

**Figure 2:**
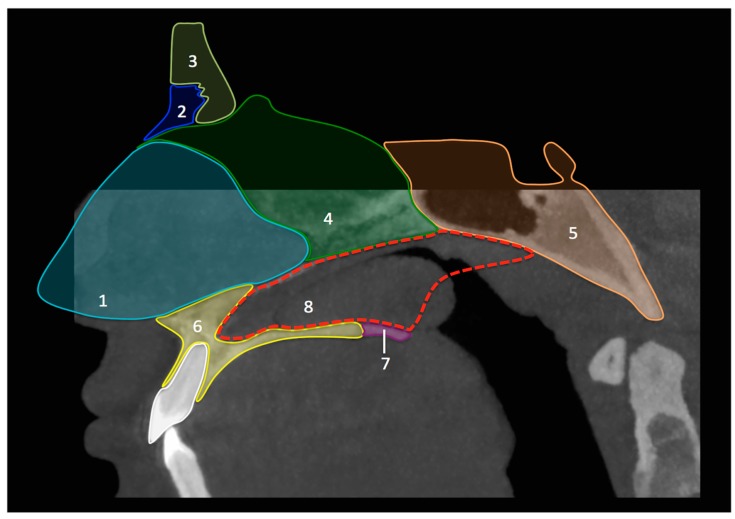
Schematic illustration representing the main anatomical elements forming the midline septum. 1. Septal cartilage; 2. Nasal bone; 3. Frontal bone; 4. Ethmoid bone (perpendicular plate); 5. Sphenoid bone; 6. Maxilla (palatine process); 7. Palatine bone (horizontal plate); 8. Vomer bone (missing).

CBCT (Figure 1) showed an incomplete nasal septum with a large postero-basal defect. There was compensatory hypertrophy of the left inferior turbinate, which protruded through the septal defect into the opposite nasal fossa and into the nasopharynx. The midline sagittal reconstructed images showed that the septal defect had a triangular shape, corresponding to the location of the missing vomer (Figure 1 and Figure 2). No other anomalies of the craniofacial region were noted, in particular no sinonasal variants and no mid-facial abnormalities.

**Figure 3:**
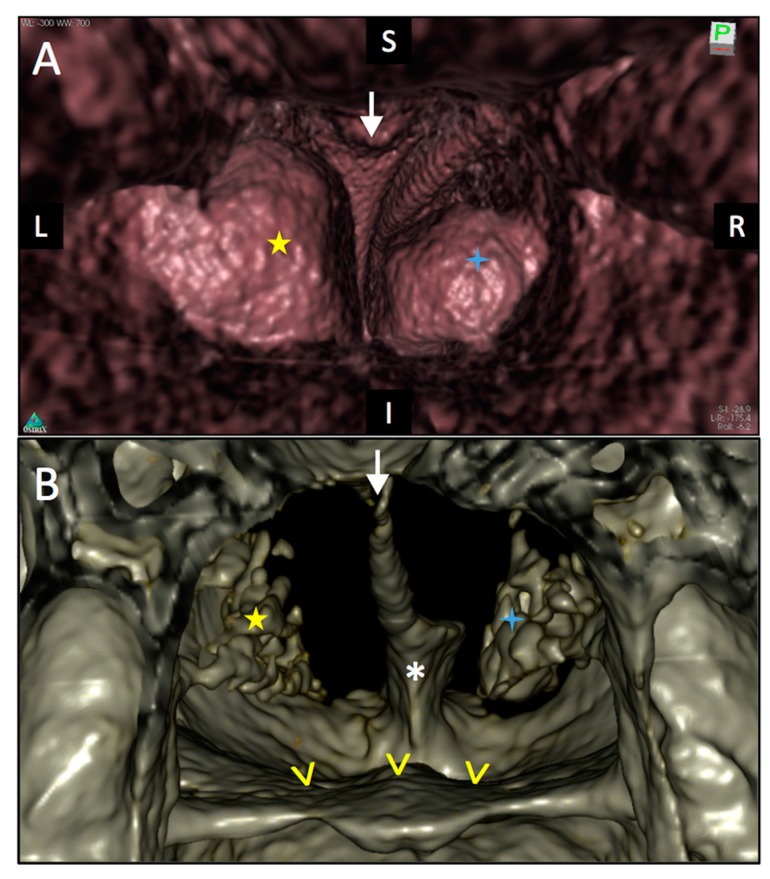
3D virtual endoscopic reconstruction (**A**) and 3D bone reconstruction (**B**) of the CBCT data set. This posterior view of the choanae shows the absence of separation between the left (L) and right (R) nasal fossae in their posterior part. Yellow star: lower left turbinate; Blue star: right lower turbinate; White arrow: Reversed triangular shape formed by the poster and inferior edges of perpendicular plate of the ethmoid bone. Yellow arrowheads: floor of the nasal fossae. White asterisk: Palatine process of the maxillary bone.

The viewer and reconstruction software OsiriX MD (V8 Pixmeo, Bernex, Switzerland) was used for image interpretation. It allowed 3D reconstruction of the facial bones and virtual endoscopic 3D reconstruction from the acquired CBCT data set (Figure 3). The posterior virtual endoscopic view of the choanae revealed the missing postero-inferior nasal septum and bulging of the hypertrophic left lower turbinate into the nasopharynx. The perpendicular plate of the ethmoid bone showed a characteristic triangular shape with a broader superior attachment to the cribriform plate and a pointed inferior bony margin.

## 3. Discussion

Nasal septum defects are most often found in patients with craniofacial malformations, as well as in patients with cleft palate [11,12]. Although deficient development of the vomer has been described as a hallmark of submucosal classic cleft palate, isolated VA is a rare anomaly reported only occasionally in the literature. Knowledge of the normal embryologic development of the os vomer is essential to understand VA [8]. The septum of the nose initially consists of a cartilaginous plate, the so-called ethmovomerine cartilage. While the posterosuperior part of the ethmovomerine cartilage ossifies forming the perpendicular plate of the ethmoid and the anteroinferior portion does not ossify (cartilaginous septum), the posteroinferior portion gives rise to the vomer. The superior ossification centers of the os vomer appear during the eighth week of gestation bilaterally. During the seventeenth gestational week, the two ossification centers unite in the midline in the caudal portion of the cartilaginous nasal septum. They form an U-shaped bone with two parallel lamellae. The septal cartilage between the two lamellae then undergoes gradual absorption. The U-shaped bony structure changes in shape and the union of the two bony lamellae extends upwards and forwards in the median plane, thereby forming the Y-shaped os vomer. These stages are critical for the development of the vomer and any interfering event can cause a defect of the postero-inferior part of the nasal septum. According to Verim et al. [11], VA can be classified into two types. In type 1 aplasia, the vomer is absent from the middle turbinate to the choanae, while in type 2 aplasia, only the caudal vomer portion is missing (partial aplasia).

Based on the embryological development of the nasal septum, several hypotheses were proposed as possible causes of congenital absence of the vomer [4,11]. The “immature ossification center” theory states that an incomplete or immature ossification center could lead to an incomplete development of the vomer. The degree of maturity of the ossification center would explain the variable shape and size of the septal defect seen in different patients. The second theory, the “incomplete downward growth” suggests that the posterior extension and downward growth of the primary nasal septum is stunted. This incomplete downward growth could, therefore, lead to a defect of the caudal end of the septum. The third theory “the incomplete touch theory” states that the incomplete union of the ossification line of the cartilaginous septum inserted in the vomerine line with the surrounding soft tissues is responsible for VA [13].

In our patient, we observed compensatory hypertrophy of the posterior left inferior turbinate. Other authors equally described lower turbinate hypertrophy in VA [5,6,9,11]. As Yilmaz et al. has speculated [5], hypertrophy may probably occur as an adaptive phenomenon in order to modulate the airflow in the nasopharyngeal region. Our patient was symptom-free, however, other studies reported that patients with VA had ENT symptoms, such as nasal obstruction, posterior nasal dripping and coughing. A reasonable explanation offered by Verim et al. suggests that vomer absence and compensatory hypertrophy of the inferior turbinates would create an environment for bacterial growth as the architectural modification would alter the airflow pressure gradient and thus may cause secretions to accumulate [11]. In addition, impaired nasal airflow may also lead to dysfunction of the Eustachian tube with resulting impaired middle ear ventilation. Although patients with nasal septal defects of traumatic, inflammatory or toxic etiology are usually treated surgically [14], as of today, surgical repair of symptomatic VA has not been described in the literature, and recommended treatment is, therefore, symptomatic.

The hereditary nature of congenital VA has been studied by Verim et al. in a case series of five patients with nasal and otologic complaints [11]. The authors evaluated the family members of five VA patients using endoscopic and radiological investigations. They detected VA in four relatives, thereby increasing the number of patients diagnosed with this anomaly from five to nine. Genetical expertise concluded that isolated VA is a multifactorial genetically transmitted disease not worth pursuing since it carries no significant medical problems [11].

Although only few cases of VA have been reported so far, several authors have suggested that the incidence of this condition may be higher than reported in the literature [4,11,12]. As CBCT is increasingly performed for a multitude of cranio-maxillofacial and dental indications, it is likely that more patients with VA will be diagnosed in the future.

## 4. Conclusions

The etiology of nasal septum defect includes nasal surgery, trauma, infection, chronic inflammatory disease, neoplasia and drug abuse. As of today, only very few cases of isolated VA have been reported in the literature. Since this anomaly is not associated with any specific clinical symptoms, VA can be easily overlooked or misinterpreted as septum perforation, especially if found incidentally on CBCT. Knowledge of the pertinent imaging features allows the correct diagnosis.
